# Eightieth Anniversary Birthday Gift

**Published:** 1919-01-25

**Authors:** 


### Eightieth Anniversary Birthday Gift.

Birthday gifts to charitable institutions are not "like leaves in Vallombrosa." Those given to celebrate the anniversary of fourscore years are always interesting. Such a gift has come to the Leicester Royal Infirmary from Mr. Charles S. Robinson, a lifelong resident and a life governor of the institution, to mark his gratitude for continued health, vigour, and activity. Mr. Robinson has long been associated with Leicester institutions. He was for many years a member of the Board of the Leicester Provident Dispensary (now taken over by the public Medical Service), and his advice was always helpful in the settlement of problems of institutional administration. He still renders useful service to the borough as one of the trustees and directors of the Leicester Savings Bank. His friends sincerely hope further years of activity and usefulness are before him.

